# Supplementary material for: Controlled Synthesis of Cs2NaYF6: Tb Nanoparticles for High-Resolution X-Ray Imaging and Molecular Detection
Source: Nanomaterials (Basel). 2025 May 12;15(10):728. doi: 10.3390/nano15100728 (PMC12114230; doi:10.3390/nano15100728)
Supplement: Supplementary file 1 [file nanomaterials-15-00728-s001.zip › nanomaterials-3647037-supplementary.pdf]

Supporting Information

Controlled synthesis of Cs_2NaYF_6: Tb nanoparticles for high-resolution X-ray imaging and molecular detection

Jian Zhao, Kunyang Wang *, Wenhui Chen, Deyang Li and Lei Lei

Institute of Optoelectronic Materials and Devices, China Jiliang University, Hangzhou 310018, China; zhaojian202411@163.com (J.Z.); 19357157434@163.com (W.C.); lideyang@cjlu.edu.cn (D.L.);
leilei@cjlu.edu.cn (L.L.)
* Correspondence: wangky@cjlu.edu.cn

Supplementary Figures

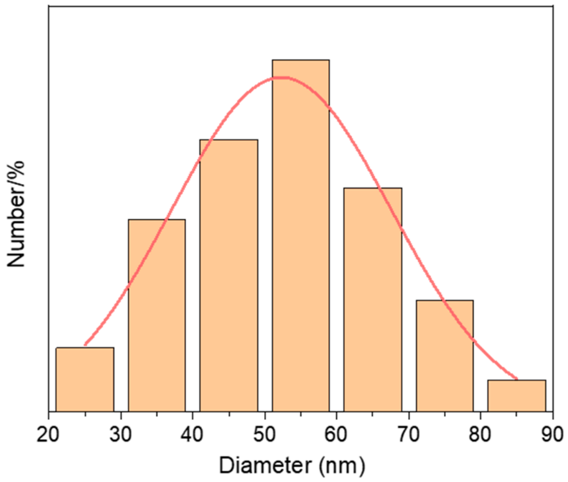

Figure S1 Histogram of size distribution of the Cs_2NaYF_6:15Tb NPs.

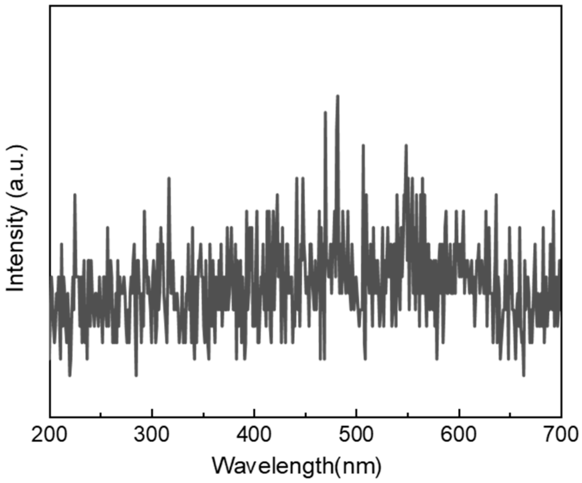

Figure S2 XEPL spectra of the Cs_2NaYF_6:Tb after continuous X-ray irradiation at 50 kV for 10 minutes.

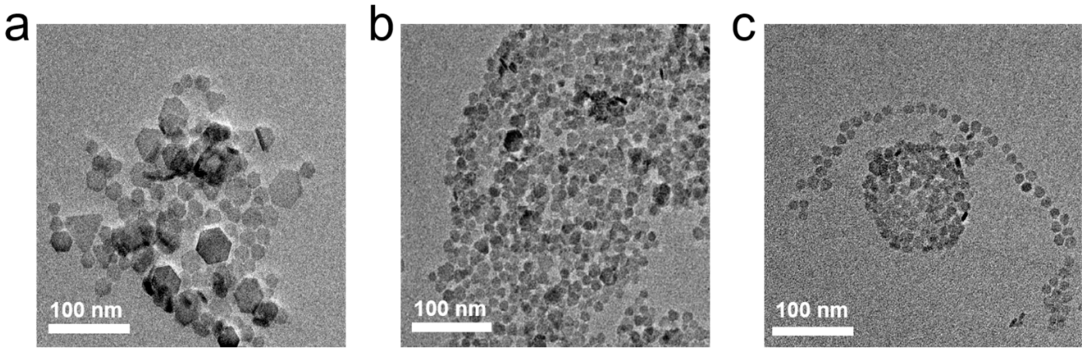

Figure S3 TEM images of the Cs_2NaYF_6:15Tb prepared with oleic acid of 13mL (a), 18 mL (b), 20 mL (c).

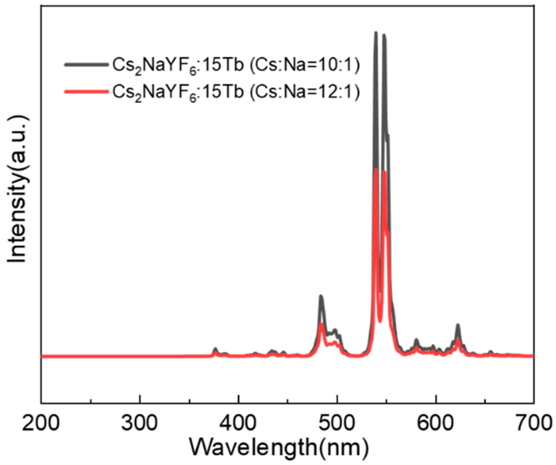

Figure S4 Comparative XEOL spectra of the Cs_2NaYF_6:15Tb ($[Cs^+]/[Na^+]$ ratio = 10:1) and Cs_2NaYF_6:15Tb ($[Cs^+]/[Na^+]$ ratio = 12:1) NPs.

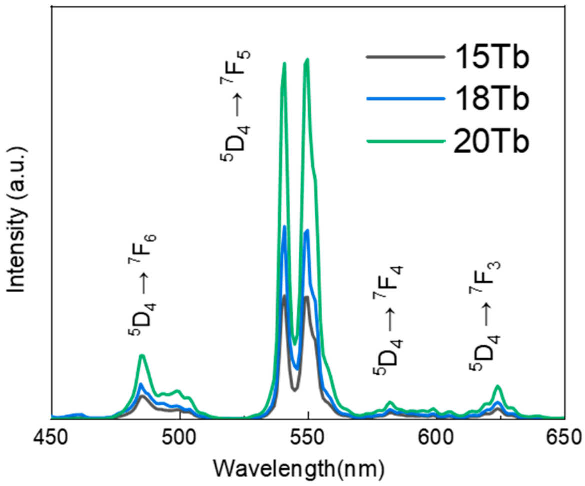

Figure S5 XEOL spectra of Cs2NaYF6:xTb (x = 15/18/20%) NPs.

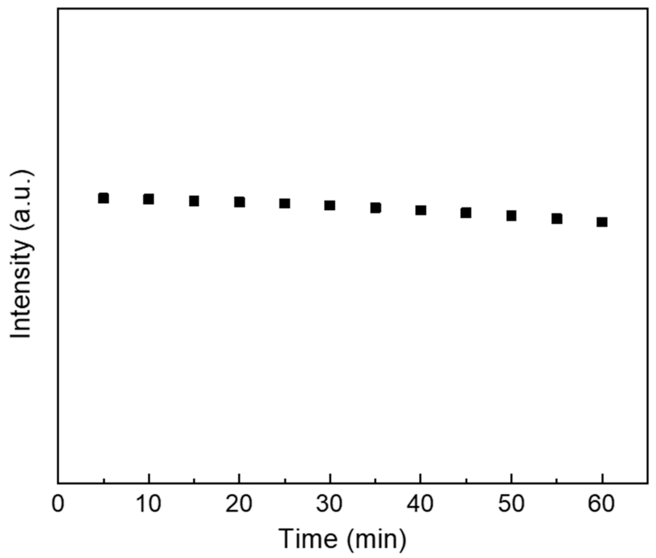

Figure S6 Intensity of Cs_2NaYF_6:15Tb NPs under X-ray irradiation over time.

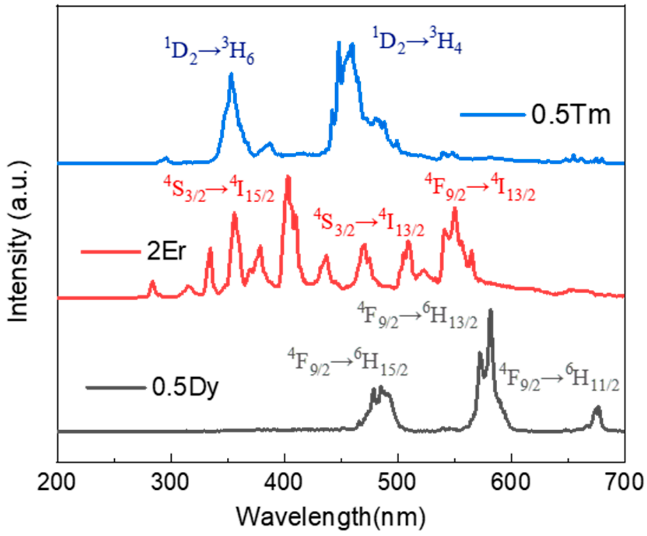

Figure S7 XEOL spectra of the Cs_2NaYF_6:0.5Tm, Cs_2NaYF_6:2Er, Cs_2NaYF_6:0.5Dy NPs.

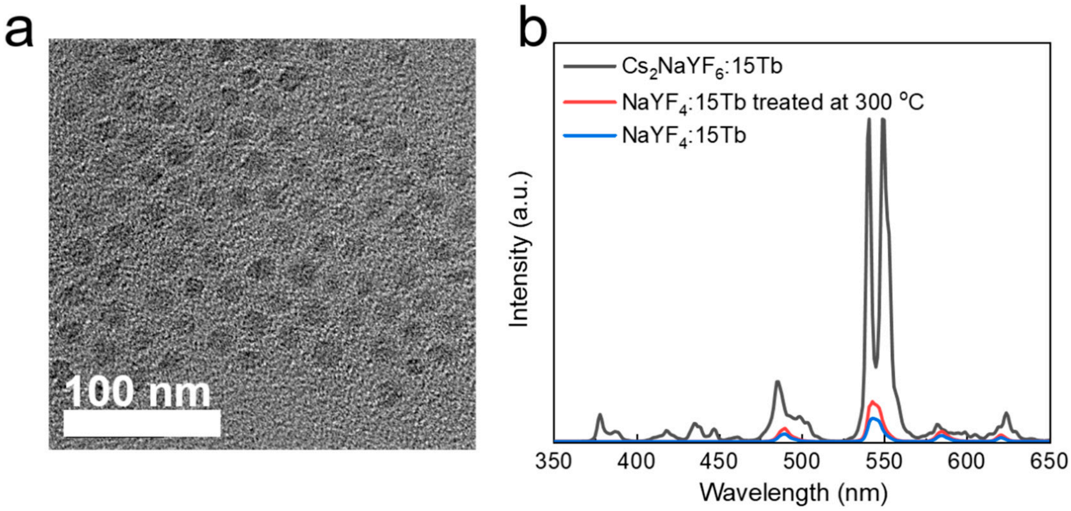

Figure S8 (a) TEM images of $NaYF_4$:15Tb NPs, (b) Comparative XEOL spectra of the $NaYF_4$:15Tb, $NaYF_4$:15Tb heat treatment at 300 ºC and Cs_2NaYF_6:15Tb NPs.

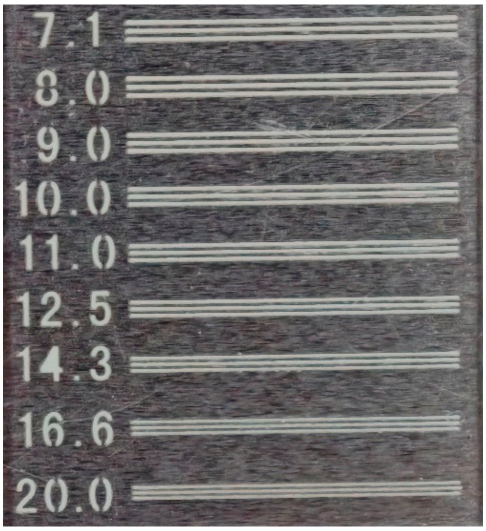

Figure S9 Image of a standard X-ray resolution test pattern.
